# Supplementary material for: Relationships among social support, decision self-efficacy, and decision regret in colorectal chemotherapy cancer patients: A mediating model
Source: PLoS One. 2026 Jan 16;21(1):e0339927. doi: 10.1371/journal.pone.0339927 (PMC12810780; doi:10.1371/journal.pone.0339927)
Supplement: Attachment — Submitted filename: Final review.docx [file pone.0339927.s002.docx]

Reviewer Notes:

**Summary of research.**

The title of the study is clearly reflecting the study’s aims. It effectively describes the significance of Colorectal cancer as a public health issue and it logically incorporates its key concepts; decision regret, social support, decision self- efficacy. The hypothesis is stated clearly and aligned with the rationale of the study. Conclusion is concise and actionable. The study is highly relevant and clinically significant to the existing scenario.

**Overall Assessment:**

**Strengths:**

Clear theoretical foundation & hypothesis. Large sample size, High internal reliability (Cronbach’s alpha >0.86). Hypotheses >2 align with mediation model.

Methodology & Results are clear and summarized accurately. The choice of statistical tests is well justified.

**Weaknesses:**

Cross sectional design is the most significant limitation.If the study is cross sectional , its conclusions must be displayed with caution, calling it a preliminary study which cannot be generalizable.

Minor typos/incomplete sentences. e.g. “accompanied by (3) missing text -----

“Lee & Bryant-Lukosius (14) “ citation mismatch with text.

Cited study by “Temitope G” lacks full reference

Hypothesis needs rephrasing as it is hard to comprehend and is weakly worded, e.g. “ Patient’s social support and self efficacy may account for

There is no mention of measurement timing of different variables checked during the course of chemotherapy – variable cycles?

Convenience sampling is fine but risks selection bias (e.g. patients in tertiary care hospitals may have better access/support)

Future directions need to be added with emphasis on multi center longitudinal study.
