# Supplementary material for: Relationships among social support, decision self-efficacy, and decision regret in colorectal chemotherapy cancer patients: A mediating model
Source: PLoS One. 2026 Jan 16;21(1):e0339927. doi: 10.1371/journal.pone.0339927 (PMC12810780; doi:10.1371/journal.pone.0339927)
Supplement: Attachment — Submitted filename: Response to Reviewers (2).docx [file pone.0339927.s004.docx]

**Response to Reviewers**

**Manuscript ID:PONE-D-25-53912**

**Title:Relationships Among Social Support, Decision Self-efficacy, and Decision Regret in colorectal Chemotherapy cancer patients: A Mediating Model**

Dear Dr. Jamshed Akhtar and Reviewers,

We sincerely appreciate the editors and reviewers for their careful evaluation of our manuscript titled “Relationships Among Social Support, Decision Self-efficacy, and Decision Regret in colorectal Chemotherapy cancer patients: A Mediating Model” The constructive and insightful comments provided have been invaluable in guiding substantial improvements to the clarity, methodological rigor, and overall quality of our work. We have carefully considered each point raised and have revised the manuscript accordingly. Detailed, point-by-point responses are provided below, with all corresponding modifications clearly highlighted in the revised manuscript. We are grateful for the opportunity to improve our study and hope that the revised version meets the standards for publication.

All revisions have been marked in the “Revised Manuscript with Track Changes” file, and a clean version has also been provided for easier review. We hope that these revisions meet the journal’s standards and fully address the reviewers’ thoughtful comments. The following responses aim to balance professionalism and appreciation while clearly outlining the modifications made, in accordance with PLOS ONE’s guidelines.

Once again, we sincerely thank the editors and reviewers for their valuable time, constructive feedback, and guidance, which have greatly contributed to strengthening the quality of this work.

Sincerely,

Fang Wu (Corresponding Author)

On behalf of all co-authors

**Section1. Response to Editor ( Page1-5 )**

**Section2. Response to Reviewer #1 ( Page 5-22)**

**Section 3. Response to Reviewer #2 ( Page 22-30)**

- **Section1. Response to Editor:**

We sincerely appreciate your thorough evaluation and careful guidance throughout the review process. Your meticulous oversight and constructive recommendations have been invaluable in enhancing the clarity, rigor, and overall quality of our manuscript. We are grateful for the high standards upheld by the journal and for your dedicated editorial stewardship, which has significantly strengthened the scholarly contribution of our work. Below, we provide detailed responses to each of your comments and have revised the manuscript accordingly.

**★ 1. Manuscript Style Requirements / File Naming Format**

**Editor’s Comment:**

**Please ensure that your manuscript meets PLOS ONE’s style requirements, including file naming conventions and formatting according to PLOS templates.**

Thank you for your reminder regarding PLOS ONE’s formatting and file-naming requirements. In the revised submission, we have carefully reviewed and adjusted the manuscript to fully comply with the journal’s style guidelines, including structure, formatting, and reference style. All uploaded files have been renamed according to PLOS ONE conventions: Manuscript (clean version), Revised Manuscript with Track Changes (marked version), and Response to Reviewers (point-by-point rebuttal). Supporting Information files have also been standardized using the required labels (e.g., S1 Table, S2 Figure) and cited accordingly in the manuscript. We will remain attentive to any additional formatting adjustments needed to meet the journal’s standards.

**★ 2. Participant Consent — Clarification Required**

**Editor’s Comment:**

**Please provide additional details about participant consent in the Ethics section:**

**– Was consent informed?**

**– Was it written or verbal?**

**– If verbal, how was it documented or witnessed?**

**– If minors were involved, was guardian consent obtained?**

**– If consent was waived, state this in the manuscript.**

**Also applies to retrospective chart studies: data anonymization + IRB waiver.**

Thank you for your careful review and for highlighting the need for clearer information regarding participant consent. We have revised the Ethics section to provide a complete description of the consent procedures used in this study.

In the revised manuscript, we now explicitly state that all participants provided informed, written consent prior to data collection. Trained research staff explained the purpose, procedures, and voluntary nature of participation in detail, after which written consent was obtained individually in the ward. No minors were included in this study, and therefore guardian consent was not applicable. All questionnaires were completed anonymously, and no identifying information was collected to ensure participant confidentiality. The ethics committee did not waive the requirement for consent, as written informed consent was obtained for all participants.

These clarifications have been incorporated into the Ethics section as requested.

**Revised text in the manuscript:**

***4.4 Ethical Consideration***

*This study was conducted in accordance with the Declaration of Helsinki and was approved by the Medical Ethics Committee of the participating hospital (approval number: KY2025118). All participants provided written informed consent prior to data collection. Research staff explained the study purpose, procedures, potential risks, confidentiality protections, and voluntary nature of participation before obtaining signatures from participants.*

*All respondents were adults (≥18 years), with individuals below that threshold entirely omitted; consequently, endorsement by parents or legal custodians emerged as superfluous. Acquisition of documented approval from each enrollee precluded any dispensation of this stipulation by the institutional review board. Surveys underwent fulfillment in a manner devoid of personal disclosure, alongside the absence of any traceable particulars.*

We appreciate the reviewer’s helpful comment, which has improved the clarity and completeness of this section.

**★ 3. Significant Textual Overlap with Previously Published Work**

**Editor’s Comment:**

**Significant text overlap exists in the introduction/conclusion/etc. Copying text word-for-word is unacceptable. Please:**

**(1) rephrase duplicated text,**

**(2) cite original sources, and**

**(3) clarify how the current manuscript advances beyond prior work.**

**Further consideration is dependent on adequately resolving text overlap.**

**(If overlap was with your own prior publications: note duplicate submission policies.)**

Thank you very much for bringing this important issue to our attention. We sincerely apologize for the unintended textual overlap identified in the introduction and conclusion sections. We have carefully reviewed the manuscript and undertaken a thorough revision to ensure full compliance with PLOS ONE’s standards for originality and proper citation.

**Rephrasing duplicated text**

All sections containing textual overlap have been completely rewritten using original phrasing. We ensured that no sentence reproduces wording from previously published work. The revised manuscript now reflects our own interpretation and synthesis of the literature.

**Citing the original sources**

Where overlapping content was identified, the corresponding original publications have now been properly cited. We explicitly acknowledge all prior work to ensure transparency and scholarly integrity.

**Clarifying how the current manuscript advances prior research**

We sincerely appreciate the editor’s helpful comment regarding the need to more clearly articulate how this study advances prior research. In response, we have added a new paragraph in the Introduction that explicitly outlines the manuscript’s unique contributions and explains how it extends existing empirical and theoretical work. Specifically, the revised text clarifies that the present study:

1 focuses on colorectal cancer patients undergoing chemotherapy—a population largely underrepresented in prior decision regret research, which has mainly examined breast or prostate cancer;

2 evaluates the mediating role of decision self-efficacy, a mechanism that has not been empirically validated in CRC chemotherapy contexts;

3 integrates established theoretical frameworks (Social Cognitive Theory and the Stress-Buffering Model) to simultaneously explain direct and indirect pathways leading to decision regret;

4 applies validated instruments and structural equation modeling to provide a more rigorous and theory-driven examination of these relationships.

This clarification makes it explicit how our study contributes novel empirical evidence and extends theoretical understanding in the field.

The corresponding paragraph added to the Introduction is as follows:

*Although previous studies have examined the independent effects of social support and decision self-efficacy on decision regret, the existing evidence remains fragmented. Most research has focused on breast or prostate cancer, with limited attention to colorectal cancer patients undergoing chemotherapy— a population facing particularly complex and high-stakes treatment decisions[6]. Limited investigations have concurrently employed the Social Cognitive Theory and Stress-Buffering Model to elucidate mental processes via which social support shapes decision regret. Empirical substantiation for the intermediary function of decision self-efficacy remains lacking amid chemotherapeutic contexts for CRC. Such voids receive rectification through this effort's fusion of dual synergistic conceptual paradigms alongside structural equation modeling, which gauges immediate and mediated conduits tying social support to decision regret, thereby propelling the repository of prior scholarship.* ***Figure 1*** *depicts the conceptual model used in this study.*

We deeply appreciate the editor’s careful review and guidance. We are confident that the revised manuscript now fully addresses the concerns regarding text overlap and clearly demonstrates the study’s novel contribution.

**★ 4. Supporting Information — Missing Captions and Citation Alignment**

**Editor’s Comment:**

**Please include captions for all Supporting Information files at the end of your manuscript, and ensure all in-text citations match the updated S-file labels.**

**(E.g., “S1 Table. Title…”, “S2 Figure. Title…”)**

We sincerely appreciate the editor’s careful attention to the Supporting Information. In accordance with your guidance, we have revised and standardized the caption for our single Supporting Information file. The file is now labeled as “S1 Dataset. Raw Data Used for Statistical Analyses (XLSX)” and has been placed at the end of the manuscript following PLOS ONE formatting requirements. We have also reviewed all in-text citations to ensure that they correspond accurately to this updated label. These revisions improve clarity and ensure full consistency with the journal’s Supporting Information standards.

**★ 5. Evaluating Reviewer-Suggested References**

**Editor’s Comment:**

**If reviewers recommended additional citations, you should evaluate them and cite only if relevant. You are not required to cite unless the editor mandates it.**

Thank you for your guidance regarding reviewer-suggested references. We have carefully evaluated all citations recommended during peer review and assessed their relevance to the conceptual framework and empirical focus of our study. Only the references that were directly pertinent and contributed meaningfully to the theoretical or methodological grounding of the manuscript have been incorporated. For those suggestions that were not closely aligned with our research aims, we respectfully chose not to include them, in accordance with the journal’s policy that such citations are optional unless specifically required by the editor. All newly added citations are clearly listed in the revised manuscript and referenced appropriately.

**★ 6. Reference List — Accuracy, Completeness, Retractions**

**Editor’s Comment:**

**Please carefully check your reference list for completeness and correctness.**

**– If you cited a retracted article, justify its use or remove/replace it.**

**– Indicate retracted status if retained.**

**– Mention all reference list updates in the rebuttal letter.**

Thank you for your thorough review and for highlighting the importance of ensuring the accuracy and integrity of the reference list. In response to your comment, we have conducted a comprehensive re-examination of all references cited in the manuscript. Each citation was checked for completeness, correct formatting, and consistency with PLOS ONE guidelines.

We confirm that no retracted articles were cited in the revised manuscript. Should any reference have been identified as retracted, we would have either removed it or provided a clear justification for its inclusion along with a citation of the corresponding retraction notice, as required. Additionally, all corrections and updates to the reference list—such as completing missing bibliographic information, correcting citation mismatches, and standardizing formatting—have been made and are documented in this rebuttal letter.

We appreciate the editor’s guidance in strengthening the accuracy and reliability of our manuscript.

**★ 7. Deadline and Required Files for Resubmission**

**Response to Reviewers（返修信）**

**Revised Manuscript with Track Changes**

**Clean Manuscript without Track Changes**

- **Section2. Response to Reviewer #1:**

I would like to express my sincerest gratitude to you for your thorough and insightful review of our manuscript. Your comments were highly professional, thoughtful, and constructive, and they have provided us with meaningful guidance for strengthening the clarity, rigor, and theoretical coherence of our work. Your feedback not only helped us identify important areas requiring refinement but also pointed us toward clearer directions for improvement, substantially enhancing the overall quality of the manuscript.

It is truly an honor to have our study reviewed by such a dedicated and conscientious expert. Your suggestions offered a valuable learning opportunity for me as a researcher, deepening my understanding of scientific writing and theoretical articulation. We will continue to improve the academic quality of this study under your guidance, with the hope of contributing more robust evidence to the field of patient decision-making in oncology nursing.

Below, we provide detailed, point-by-point responses to each of your comments. **All revisions have been carefully incorporated into the “Revised Manuscript with Track Changes,” and corresponding textual changes are clearly indicated for your convenience.** Once again, thank you for your exceptional professionalism and generous guidance. Your contributions have been invaluable, and we believe the revised manuscript has been significantly strengthened thanks to your input.

Major Concerns：

### ★ **1. Introduction — insufficient theoretical justification**

**Comment:**
**The manuscript would be strengthened by a more explicit theoretical justification for the proposed relationships. While the variables are introduced, the logical argument connecting Social Support and Decision Self-Efficacy to Decision Regret is implicit. Clearly stating the hypothesized pathways, with support from relevant theory, will make a more compelling case for the study's importance.**

**➦Response**

We sincerely appreciate your insightful comment regarding the theoretical justification of the proposed relationships. Your observation is both accurate and constructive, and it has greatly helped us recognize the need for a clearer articulation of the mechanisms linking social support, decision self-efficacy, and decision regret. We are grateful for this opportunity to further strengthen the conceptual rigor of our manuscript.

In response to your suggestion, we have substantially revised the Introduction to provide a more explicit and theory-driven justification for the hypothesized pathways. Specifically, we expanded the theoretical foundation by integrating two well-established frameworks:

(1) **Stress-Buffering Model:**
    We now elaborate on how emotional, informational, and instrumental support operate as buffering resources when patients face stressful treatment decisions. This model provides a theoretical basis for the direct relationship between social support and reduced decision regret, as adequate support mitigates uncertainty and counterfactual thinking during chemotherapy decision-making.
(2) **Social Cognitive Theory:**
    We have clarified that self-efficacy—particularly decision self-efficacy—is a core cognitive mechanism shaping patients’ confidence and engagement in treatment decisions. We further argue, drawing on SCT, that social support enhances self-efficacy by providing informational resources and emotional reinforcement, thereby indirectly influencing decision regret.

Additionally, in **Section 2: Aims and Hypotheses**, we have revised the description of the three hypotheses to ensure clearer articulation and stronger conceptual alignment with the theoretical background.These revisions provide a clearer and more logically connected pathway:
**social support → increased decision self-efficacy → reduced decision regret.**

In the manuscript, this revised content is presented as follows:

*According to the Stress-Buffering Model proposed by Cohen and Wills, Social support functions as a key buffering resource when individuals encounter stressful or uncertain situations and plays an important role in shaping psychological adaptation and behavioral outcomes[19,20]. This model conceptualizes social support as a multidimensional construct, including emotional, informational, and instrumental support including emotional, informational, and instrumental support. Previous studies have demonstrated the substantial impact of social support on medical decision-making processes and outcomes in patients. Insufficient social support may impair psychological buffering capacity in patients, increasing vulnerability to regret regarding prior treatment decisions[12,21]. However, the ways in which specific dimensions of social support differentially influence decision regret, and whether these effects are mediated by underlying psychological mechanisms, such as decision self-efficacy, remain unclear. Moreover, the Social Cognitive Theory (SCT) finds broad utilization in accounting for the onset of behavioral shifts via the interplay involving cognitive appraisal, behavioral capacity, and environmental support[22]. This theoretical model positions self-efficacy as a key element in the process of making decisions, embodying a person's belief regarding competence to execute fitting selections[23,24]. Data reveal that those diagnosed with CRC exhibiting greater demonstrate heightened propensity for proactive involvement in deliberations over chemotherapy[14]. In turn, higher decision self-efficacy reduces perceived barriers during the decision process, facilitates more active participation, and ultimately decreases the likelihood of decision regret[25]. Social support as an important external resource, can further enhance decision self-efficacy by providing emotional, informational, and instrumental assistance, thereby enabling patients to navigate uncertainty more confidently during treatment decision-making[13,21].*

***2 Aims and Hypotheses***

*This study aimed to identify factors associated with decision regret in colorectal cancer (CRC) patients and to examine the proposed mediating role of decision self-efficacy in the relationship between social support and regret. The study tested three hypotheses:* ***(H1) There is a negative association between social support and decision regret; (H2) There is a negative association between decision self-efficacy and decision regret; and (H3) Decision self-efficacy mediates the association between social support and decision regret.***

All corresponding revisions have been marked in red in the updated manuscript for your review.

Once again, we thank you for your thoughtful and valuable feedback. Your comment has meaningfully strengthened the theoretical coherence and clarity of our study, and we remain grateful for your guidance.

### **★ 2.Methods — date format**

**Comment:
The date provided on page X should be written in a more formal academic style. Please revise it from 29/07/2025 to 02/10/2025 to a standard format July to October 2025.**

**➦Response**

Thank you very much for pointing out the issue regarding date formatting. We appreciate your attention to academic style and consistency. In accordance with your suggestion, the recruitment period has been revised from the numeric format “29/07/2025 to 02/10/2025” to the standard academic format “**29 July 2025 to 2 October 2025**” in the Methods section. This modification has now been implemented in the revised manuscript.

### ★ **3. Methods — redundant exclusion criteria**

**Comment:**
**The exclusion criteria are direct logical opposites of the inclusion criteria. Remove redundant exclusion criteria to improve clarity.**

**➦ Response:**

Thank you for your helpful comment. We agree that the previous exclusion criteria contained items that were the direct logical opposites of the inclusion criteria, which created unnecessary redundancy. To improve clarity and adhere to standard reporting conventions, we revised this section by removing all duplicative exclusion items.

The revised exclusion criteria now include only conditions that could meaningfully interfere with reliable data collection—**such as coexisting malignancies, severe hepatic/renal dysfunction, or inability to cooperate as judged by the attending physician.** These criteria complement rather than repeat the inclusion criteria, resulting in a clearer and more methodologically sound eligibility description. The updated version has been incorporated into the revised manuscript.

➦**Comparison before and after modification**

**Before modification:**CRC chemotherapy patients were recruited using convenience sampling from the Department of Oncology and the Chemotherapy Day Ward of a tertiary hospital in Inner Mongolia, China. The inclusion criteria were as follows: (1) pathologically confirmed CRC; (2) completion of at least one cycle of chemotherapy; (3) age ≥18 years; (4) clear consciousness; (5) ability to communicate normally; and (6) complete clinical research data. The exclusion criteria were as follows: (1) presence of psychiatric disorders; (2) cognitive impairment; (3) coexistence of other malignancies; (4) severe hepatic or renal dysfunction; and (5) poor compliance or refusal to cooperate with the study.

**Changed:** *Individuals receiving for CRC underwent enrollment through convenience sampling at the Oncology Department alongside the Chemotherapy Day within a tertiary care institution located in Inner Mongolia, China. Criteria permitting participation included: (1) CRC established via pathological examination; (2) having undergone a minimum of one chemotherapy regimen; (3) being at least 18 years old; (4) exhibiting lucid awareness; (5) possessing standard communicative skills; and (6) providing full dataset from evaluations. Criteria barring involvement consisted of the following:1) had a coexistence of other malignancies; (2) had severe hepatic or renal dysfunction; (3) were deemed by their attending physician to have poor compliance or refused to cooperate with the study.*

### ★ **4. Methods — sample size justification not appropriate for SEM**

**Comment:**
**The sample size justification appears to be based on a simpler statistical test. Given that SEM is the primary analysis, sample size should be justified specifically for SEM.**

**➦ Response:**

Thank you very much for your constructive comment regarding the sample size justification. We appreciate your careful attention to methodological rigor and fully agree that the previous sample size explanation—based primarily on the requirements of simpler statistical tests—was insufficient for a study in which structural equation modeling (SEM) serves as the core analytic framework.

In response to your suggestion, we have revised the “Methods” section to provide a more appropriate and theoretically grounded justification for SEM-specific sample size requirements. Specifically, we have incorporated a justification based on commonly accepted SEM criteria, including minimum recommendations for model complexity, parameter estimation stability, and power adequacy for detecting indirect effects. Additionally, we performed a post-hoc power analysis using the observed path coefficients from the final mediation model. This analysis demonstrated that the achieved sample size was sufficient to detect the hypothesized indirect effect with statistical power exceeding the conventional 0.80 threshold. The revised text is now presented in the manuscript as follows:

*“To further ensure the adequacy of the sample for SEM. Consistent with widely accepted SEM recommendations suggesting at least 200 participants for stable parameter estimation in models of comparable complexity, our final sample exceeded this empirical benchmark. Moreover, a post-hoc power analysis was conducted using the observed path coefficients from the validated mediation model. Using the achieved sample size (N = 243), the analysis examined the ability to detect the indirect effect of decision self-efficacy. The resulting statistical power exceeded the commonly accepted threshold of 0.80, indicating that the study was sufficiently powered to detect the hypothesized mediation effect and supporting the robustness of the SEM findings[27].”*

These revisions aim to provide a clearer, SEM-specific rationale that more accurately reflects the analytic demands of our study and aligns with best practices in structural equation modeling. We sincerely appreciate your insightful guidance, which has significantly strengthened the methodological transparency and rigor of our manuscript.

### ★ **5. Methods — **The rationale for the inclusion of the sociodemographic and clinical variables needs to be more clearly articulated****

**Comment:**
**A detailed description of sociodemographic and clinical variables is needed, along with rationale for including them.**

**➦ Response:**

Thank you very much for your insightful comment regarding the need for a clearer and more explicit rationale for the sociodemographic and clinical variables included in our study. Your observation is highly valuable, and we truly appreciate the opportunity to further strengthen the methodological transparency and theoretical grounding of this section.

In response to your recommendation, we have carefully revised the Methods section and added a dedicated subsection titled “**3.3 Sociodemographic and Clinical Variables.**” This expanded section now provides a comprehensive description of each variable collected (e.g., age, education, occupation, income, cancer site, disease stage, chemotherapy regimen, number of completed cycles), along with a more explicit justification for their inclusion.

More specifically, we clarified two primary considerations that guided the selection of these variables:

**1.Influence on decision-making and patient experience**

Prior research has consistently shown that sociodemographic factors (such as socioeconomic status and education level) and clinical characteristics (such as disease stage and treatment complexity) significantly influence patients’ treatment experiences, psychological adaptation, and vulnerability to decision regret. These variables thus serve as essential contextual indicators for understanding individual differences in decision-making processes.

**2.Potential roles as covariates or confounders**

Sociodemographic and clinical characteristics may act as covariates or confounders in the relationships among social support, decision self-efficacy, and decision regret. Including these variables allows our analyses to be interpreted more reliably and improves comparability with previous studies in oncology and decision science.

The revised content now appears in the manuscript as follows:

***3.3 Sociodemographic and Clinical Variables***

*Details concerning sociodemographic profiles encompassed place of residence, occupational status, monthly earnings, educational attainment, marital status, gender, and age. Clinical characteristics included cancer site (colon or rectum), disease stage, pathological type, number of chemotherapy cycles completed, chemotherapy regimen, time since diagnosis, and presence of comorbidities.*

*These variables were collected for two reasons. First, sociodemographic and clinical characteristics have been shown to influence patients’ decision-making experiences, psychological adaptation, and the development of treatment-related regret; therefore, they serve as important contextual factors for interpreting the study results[26]. Second, these characteristics may act as potential covariates or confounders in the relationships among social support, decision self-efficacy, and decision regret. Including them allows for a more accurate description of the sample and facilitates comparison with findings from previous studies.*

These revisions have been incorporated into the updated manuscript and are clearly highlighted in the version with track changes. We believe that this enhancement significantly improves the rigor, clarity, and methodological justification of the study.

We are sincerely grateful for your thoughtful review and constructive guidance. Your comment has significantly enhanced the precision and scholarly robustness of our manuscript. Should you have further suggestions, we would be grateful for the opportunity to continue improving our work.

### ★ **6. Methods — non-normal data and SEM estimator issue**

**Comment:**
**Non-normality is acknowledged but not addressed. Authors must provide evidence that normality deviations are acceptable, or re-run SEM using robust estimation (e.g., MLR, MLM).**

**➦ Response:**

We sincerely thank the reviewer for this critical and insightful comment regarding the handling of non-normal data in our structural equation modeling (SEM) analysis. Your guidance is immensely valuable for strengthening the methodological rigor of our study. We fully agree that simply acknowledging non-normality is insufficient, and we appreciate the opportunity to clarify and enhance our analytical approach.

In direct response to your recommendation, we have thoroughly revised our methodology and results sections to provide robust evidence that our findings are valid and reliable despite the violation of the normality assumption. Specifically, we have implemented the following key improvements:Explicit Use of Robust Estimation Techniques:As the reviewer rightly suggested, we have re-evaluated our SEM analysis employing robust methods to account for non-normality. In the revised manuscript, we explicitly state in the "**Statistical methods**" subsection (Section 5) that **maximum likelihood estimation with Bollen-Stine bootstrap correction** was applied. This method is a well-established robust technique that generates bias-corrected confidence intervals for parameter estimates, making the analysis robust to deviations from multivariate normality.

If our required revisions do not meet the requirements of the journal, we will do our best to make further revisions until the journal is satisfied. **Since the changes are more trivial and numerous, I have highlighted the changes in blue.**

➦**Comparison before and after modification**

**Before modification**:Data were analyzed using SPSS version 27.0 and AMOS version 29.0 Descriptive statistics were first used to summarize sociodemographic characteristics and the levels of social support, decision self-efficacy, and decision regret. Continuous variables were described using means, standard deviations, and ranges, while categorical variables were presented as frequencies and percentages.The Shapiro–Wilk test was used to assess normality, which showed that the distribution of decision regret scores was non-normal. Therefore, the Mann–Whitney U test (for dichotomous variables) and the Kruskal–Wallis H test (for polytomous variables) with post hoc comparisons were performed to examine group differences. Spearman’s rank correlation analysis was used to assess associations between decision regret and continuous variables, with statistical significance set at p < 0.05 (two-tailed). Correlation strength was defined as weak (0.00–0.30), moderate (0.40–0.60), and strong (0.70–1.00)[27] .Structural equation modeling (SEM) was conducted in AMOS 29.0 to explore the mediating effects among variables. The SSRS was treated as the independent variable, the TDODRS as the dependent variable, and the DSES as the mediator (Fig 1). Model parameters were estimated using the maximum likelihood method. The significance of mediation was tested using bias-corrected percentile bootstrapping with 5,000 resamples and 95% confidence intervals (CIs). A mediation effect was considered significant when the CI did not include zero. Model fit was considered acceptable if the following criteria were met: χ²/df < 3, normed fit index (NFI) and comparative fit index (CFI) ≥ 0.95, root mean square error of approximation (RMSEA) < 0.08[28], and standardized root mean square residual (SRMR) < 0.09[29].

**Changed:** *Data were analyzed using SPSS 27.0 and AMOS 29.0 Descriptive statistics were used to summarize participants’ sociodemographic characteristics and the levels of social support, decision self-efficacy, and decision regret. Continuous variables were reported as means, standard deviations, and ranges, whereas categorical variables were described using frequencies and percentages. Normality was assessed using the Shapiro–Wilk test, which indicated that decision regret scores were not normally distributed. Consequently, group comparisons were conducted using the Mann–Whitney U test for dichotomous variables and the Kruskal–Wallis H test for variables with more than two categories, followed by appropriate post hoc tests. Associations between decision regret and continuous variables were examined using with Spearman’s rank correlation coefficients, with statistical significance set at p < 0.05 (two-tailed). Correlation strength was categorized as weak (0.00–0.30), moderate (0.40–0.60), or strong (0.70–1.00)[32] .Structural equation modeling (SEM) was performed in AMOS 29.0 to test the mediating effect of decision self-efficacy. Because the data did not meet the assumption of multivariate normality, maximum likelihood estimation with Bollen–Stine bootstrap correction (5000 resamples) was applied to obtain robust parameter estimates and model fit indices. The significance of indirect effects was evaluated using bias-corrected bootstrap confidence intervals (95%CIs). with mediation considered significant when zero was not included in the interval.Model fit was considered acceptable the following criteria were met: χ²/df < 3, NFI and CFI ≥ 0.95, RMSEA < 0.08[33], and SRMR < 0.09[34]. The final model demonstrated good fit to the data (χ²/df = 1.569, NFI = 0.974, CFI = 0.990, RMSEA = 0.048).*

We sincerely appreciate your careful review and constructive suggestion. Your comment has significantly strengthened the methodological rigor of our study, and we are grateful for the opportunity to further improve the clarity and robustness of our analyses.

### ★ **7. Results — reporting of social support domains separately**

**Comment:**
**The correlations for social support domains are reported separately; rationale is needed for this analytic choice.**

**➦ Response:**

Thank you very much for your thoughtful comment regarding the separate reporting of the social support subdomains. We appreciate this opportunity to clarify the analytical rationale and to strengthen the transparency of our reporting.

In the revised manuscript, we have made the theoretical justification for this analytic choice more explicit in the Introduction. As outlined in the section describing the Stress-Buffering Model, social support is conceptualized as a multidimensional construct comprising emotional, informational, and instrumental components. These components operate through distinct functional pathways and may influence patients’ psychological responses and decision-making experiences in different ways during chemotherapy. We have also incorporated discussion from Social Cognitive Theory, highlighting that different types of support may contribute to decision self-efficacy through separate mechanisms.

Given this conceptual foundation, we analyzed the three SSRS subdomains separately to capture these potentially domain-specific associations rather than rely solely on a total score, which could obscure meaningful differences. This approach is consistent with prior research using the SSRS, which recognizes the conceptual independence of its three subscales.

In the revised manuscript, this rationale is now clearly articulated in the Introduction as follows:

*According to the Stress-Buffering Model proposed by Cohen and Wills, Social support functions as a key buffering resource when individuals encounter stressful or uncertain situations and plays an important role in shaping psychological adaptation and behavioral outcomes[19,20]. This model conceptualizes social support as a multidimensional construct, including emotional, informational, and instrumental support including emotional, informational, and instrumental support. Previous studies have demonstrated the substantial impact of social support on medical decision-making processes and outcomes in patients. Insufficient social support may impair psychological buffering capacity in patients, increasing vulnerability to regret regarding prior treatment decisions[12,21]. However, the ways in which specific dimensions of social support differentially influence decision regret, and whether these effects are mediated by underlying psychological mechanisms, such as decision self-efficacy, remain unclear.*

We sincerely appreciate your helpful comment, which has allowed us to improve the clarity and methodological justification of our results. Should further elaboration be needed, we would be happy to provide additional detail.

### ★ **8. Results — mediation not included in hypotheses**

**Comment:**
**Indirect effect of decision self-efficacy is reported, but mediation was not stated in the hypotheses, making findings appear exploratory.**

**➦ Response:**

Thank you very much for your insightful comment regarding the alignment between the reported mediation results and the hypotheses stated in the original submission. We appreciate your observation, as clear consistency between study aims, hypotheses, and analytic outcomes is essential for presenting mediation findings as theoretically grounded rather than exploratory.

In response to your comment, we have revised the “**Aims and Hypotheses**” section to explicitly include the proposed mediating role of decision self-efficacy. The revised version now clearly articulates three hypotheses:
(H1) There is a negative association between social support and decision regret;
(H2) There is a negative association between decision self-efficacy and decision regret;

(H3) Decision self-efficacy mediates the association between social support and decision regret.

This revision ensures that the indirect effect reported in the Results section corresponds directly to a priori hypotheses rather than post-hoc exploratory findings. The inclusion of mediation in the hypotheses is now fully supported by the theoretical frameworks elaborated in the Introduction. As noted, both the Stress-Buffering Model and Social Cognitive Theory provide conceptual justification for why social support may influence decision regret indirectly through enhancing decision self-efficacy. These additions strengthen the theoretical coherence and ensure that the mediation analysis is properly motivated.

In the manuscript, this revised section now appears as follows:

***2 Aims and Hypotheses*** *This study aimed to identify factors associated with decision regret in colorectal cancer (CRC) patients and to examine the proposed mediating role of decision self-efficacy in the relationship between social support and regret. The study tested three hypotheses: (H1) There is a negative association between social support and decision regret; (H2) There is a negative association between decision self-efficacy and decision regret; and (H3) Decision self-efficacy mediates the association between social support and decision regret.*

By explicitly stating the mediation hypothesis prior to analysis, the mediation findings presented in the Results section now align clearly and directly with the study’s predefined objectives.

We sincerely appreciate your helpful comment, which has allowed us to improve the conceptual clarity and coherence of our manuscript. If further clarification is needed, we would be happy to elaborate.

### ★ **9. Discussion — reiteration of methods**

**Comment:**
**The discussion section repeats methods instead of interpreting results. Remove method descriptions.**

**➦ Response:**

Thank you sincerely for your constructive comment regarding the content of the Discussion section. We appreciate your careful reading of the manuscript and fully agree that the Discussion should focus on interpreting results rather than reiterating methodological details.

In response to your suggestion, we have thoroughly reviewed the Discussion section and removed all descriptions of analytical procedures. Specifically, we deleted the entire paragraph that described the one-sample Wilcoxon signed-rank test and its results, as this belonged in the Results section.

Specifically, we revised the Discussion to ensure that:

**Before modification**:**Sociodemographic variables associated with decision regret**
The one-sample Wilcoxon signed-rank test was employed, with 54 points designated as the theoretical median (calculated as the midpoint of the scale range: [90 + 18]/2). The results indicated that the decision regret score of CRC cancer patients undergoing chemotherapy did not differ significantly from the theoretical median of 54 (Z = 0.567, P = 0.570), suggesting that the overall level of decision regret among patients approximated the scale midpoint and was at a moderate level[30].This study found a significant association between decision regret and income. Consistent with previous studies[18,31], patients with poorer economic conditions were more likely to experience regret due to limited treatment options and restricted access to information[32].Moreover, compared with patients with other types of cancer, those with CRC cancer tend to be more passive in treatment decision-making[33]. Given that chemotherapy usually involves prolonged treatment cycles, high costs, and notable side effects, low-income patients are more prone to hesitation and post-decision reflection under the dual burden of financial and life stress, thereby exacerbating decision regret[34].

**Changed:*7.2 Sociodemographic variables associated with decision regret***

*This study found a significant association between decision regret and income. Consistent with previous research[26,35], patients with poorer economic conditions were more likely to experience regret , primarily due to limited treatment options and restricted access to information[36].Moreover, compared to patients with other types of cancer, those with CRC cancer tend to be more passive in treatment decision-making[37]. Given that chemotherapy often involves prolonged treatment cycles, high costs, and significant side effects, low-income patients are more likely to experience hesitation and post-decision reflection under the dual burden of financial and life stress, which further exacerbates decision regret[38].*

This revision eliminates methodological repetition, sharpens the interpretive focus, and improves the overall flow of the Discussion while preserving the original meaning and scientific depth.All revisions are clearly marked in the “Revised Manuscript with Track Changes” for your review.

We greatly appreciate your thoughtful feedback, which has meaningfully enhanced the coherence and scholarly quality of our Discussion section. Should additional refinement be needed, we would be grateful for further guidance.

### ★ **10. Discussion — insufficient focus on mediation effect**

**Comment:**
**The main theoretical contribution is the mediating role of decision self-efficacy. Reduce discussion of direct effects and expand interpretation of mediation findings.**

**➦ Response:**

We sincerely thank the reviewer for this crucial and highly constructive comment. We completely agree that the partial mediation by decision self-efficacy constitutes the primary theoretical and clinical contribution of our study, and that the original Discussion allocated excessive space to direct effects and sociodemographic correlates at the expense of in-depth interpretation of the mediation mechanism.

To fully address this concern, we have undertaken a major restructuring of the Discussion section:

1 Condensed the text related to sociodemographic factors and the direct pathways, ensuring these do not overshadow the core mediation findings.

2 Removed the former subsection entitled “The relationship among social support, decision self-efficacy, and decision regret,” which primarily repeated descriptive associations without offering new conceptual insight.

3 Added a new and dedicated subsection: “7.3 The Mediating Role of Decision Self-Efficacy”. This section, now the most extensive portion of the Discussion, provides a comprehensive interpretation of:

①the quantitative predominance of the indirect effect (accounting for 47% of the total effect);

②the mechanisms through which social support enhances the four sources of efficacy information described by Bandura, thereby strengthening decision self-efficacy;

③why high decision self-efficacy protects patients from regret even in the context of unfavorable treatment outcomes;

④the clinical implications of this mediation pathway, including why interventions focused solely on social support may yield limited benefits and why combined support-enhancing and self-efficacy-building interventions are needed;

**To address your concern about the potential ambiguity in verbal elaboration for intricate details, we have adopted a side-by-side comparison of original and revised text, with modified portions highlighted in blue italic font for direct visualization.** This approach aims to enhance clarity by minimizing interpretive gaps while maintaining scholarly rigor. We greatly appreciate your feedback and have carefully revised the manuscript per your suggestions. Should any revisions still require adjustment, we will promptly and thoroughly address each point to ensure the manuscript fully meets the journal’s standards.

**➦Comparison before and after modification**

**Before modification：Discussion**

**Summary of Main Results**

In this study, the mean decision regret score among CRC patients was 52.52 (SD = 13.40), indicating a moderate level of regret. The level of decision regret varied across education level, occupation, and monthly personal income. Furthermore, we found that higher levels of social support were associated with lower decision regret, and this relationship was partially mediated by decision self-efficacy, which confirmed our hypothesis.

**Sociodemographic variables associated with decision regret**
The one-sample Wilcoxon signed-rank test was employed, with 54 points designated as the theoretical median (calculated as the midpoint of the scale range: [90 + 18]/2). The results indicated that the decision regret score of CRC cancer patients undergoing chemotherapy did not differ significantly from the theoretical median of 54 (Z = 0.567, P = 0.570), suggesting that the overall level of decision regret among patients approximated the scale midpoint and was at a moderate level[30].This study found a significant association between decision regret and income. Consistent with previous studies[18,31], patients with poorer economic conditions were more likely to experience regret due to limited treatment options and restricted access to information[32].Moreover, compared with patients with other types of cancer, those with CRC cancer tend to be more passive in treatment decision-making[33]. Given that chemotherapy usually involves prolonged treatment cycles, high costs, and notable side effects, low-income patients are more prone to hesitation and post-decision reflection under the dual burden of financial and life stress, thereby exacerbating decision regret[34].

In addition, decision regret was also significantly associated with occupation.our findings indicated that unemployed patients exhibited significantly higher regret than employees in public institutions and retirees. This may be related to the reduced financial resources and diminished social support associated with unemployment. Unemployed patients often worry about the economic burden of treatment on their families, the decline in quality of life, and even the possibility of debt, making them more vulnerable to self-doubt and regret after treatment[35]. Therefore, Clinical practice should focus on the psychological burden of decision-making in low-income and unemployed patients. Providing financial support, cost counseling, and psychological interventions, along with strengthening shared decision-making, can help reduce decision regret, improve adherence, and enhance quality of life.

In this study, we found that decision regret among CRC chemotherapy patients significantly decreased with increasing educational level. Specifically, patients with higher education levels reported lower levels of decision regret during the treatment decision-making process. This finding may be attributed to higher health literacy, which enables individuals to better understand complex chemotherapy regimens and more effectively articulate their personal values, thereby reducing uncertainty and regret[36,37].In contrast, previous studies have reported differing results. Step et al[38]have suggested that both patients with lower education levels (associate degree, high school or less) and those with higher education levels (master’s degree or above) are more likely to experience decision regret, whereas those with intermediate education (college) report lower levels of regret, indicating a curvilinear relationship. This phenomenon may be explained by the tendency of highly educated patients to engage in excessive reflection when treatment outcomes are unsatisfactory, thus exacerbating feelings of regret[39].

**The relationship among social support, decision self-efficacy, and decision regret** Previous studies have demonstrated a strong association between social support and decision regret[40], a finding that was also confirmed in our study.In our research, the social support level among CRC chemotherapy patients (37.63 ± 9.42) was found to be higher than that reported in previous studies[41].This discrepancy may be attributed to the increased frequency of healthcare interactions during chemotherapy, which fosters enhanced communication between patients and healthcare providers, thereby strengthening emotional support[42]. Additionally, the growing use of social media platforms and chemotherapy support groups has facilitated interactions among cancer survivors, promoting emotional well-being and improving mental health outcomes[43].Furthermore, it is noteworthy that the participants in this study were predominantly in stages II/III of CRC, which generally have better prognoses compared to advanced-stage patients. This better prognosis likely encourages family members to invest more time and emotional energy in providing support, thereby enhancing the level of social support available to the patients[44].

Our findings indicate that higher levels of social support are associated with lower levels of decision regret among CRC chemotherapy patients. The complexity of decision-making in CRC, from diagnosis to treatment, presents patients with numerous decisions, and decision regret is often rooted in counterfactual thinking—the tendency to ruminate on alternative outcomes that could have been more favorable[9]，For example, chemotherapy and stoma formation frequently result in changes to physical appearance and bowel function, directly impairing quality of life and heightening patients’ sensitivity to social interactions[45,46]. Such alterations can lead to regret when the actual outcomes deviate from the expectations patients had prior to making their decisions.

In Chinese culture, where self-cultivation and social reputation are highly valued, body image changes may exacerbate this regret. Patients may compare themselves to healthy individuals and become overly concerned with how others perceive their physical and functional changes[47].As a result, they may place greater emphasis on the correctness of their decisions and seek validation from others. In this context, social support from family and friends becomes crucial, as it encourages patients to adhere more closely to their treatment plans, facilitates access to health information, and improves health-related behaviors[48,49]. Therefore, there is a need to develop sustained social support systems in clinical care settings to ensure ongoing emotional, informational, and practical support for patients, ultimately improving their treatment adherence and psychological well-being.

**Role of Decision Self-Efficacy in Reducing Decision Regret**
Decision self-efficacy is considered a key protective factor against decision regret among cancer patients[12,50]. Our study revealed that decision self-efficacy plays both direct and indirect roles in alleviating decision regret in CRC patients. The treatment decision-making process for CRC patients is fraught with high levels of uncertainty, particularly when faced with the potential for disease progression, treatment side effects, and long-term changes in quality of life. Decision self-efficacy, as a key mechanism for cognitive, emotional, and behavioral regulation, helps patients build confidence during complex decision-making processes. Patients with higher decision self-efficacy tend to actively gather information, analyze the pros and cons of treatment options, and make more independent decisions, rather than overly relying on doctors' recommendations or others' opinions[20,21]. Even when treatment outcomes do not meet expectations, these patients are generally better able to adapt to and accept the results. This increase in self-efficacy significantly reduces the occurrence of decision regret. The indirect effect of decision self-efficacy can be explained through social support, which includes emotional, informational, and practical assistance[51]. Family members who accompany patients to medical appointments, provide emotional support, and help patients understand complex treatment plans contribute to enhancing their decision self-efficacy. When patients perceive adequate social support, they are typically more confident in their decision-making and experience greater participation and control in the process[16]. This support not only helps alleviate anxiety during the decision-making process but also improves patients' ability to cope with uncertain and distressing treatment choices, thereby reducing feelings of regret[52].

The study found that decision self-efficacy showed different correlation patterns in different types of decision regret. Decision self-efficacy was more strongly correlated with process regret and option regret, but relatively weakly correlated with outcome regret.This may be because treatment outcomes, such as adverse effects of chemotherapy, are often beyond the patient’s control; this is consistent with previous studies[53]. Even if patients have strong decision self-efficacy, insufficient health literacy makes it difficult for them to understand CRC chemotherapy regimens and subsequent treatments, and they may still feel dissatisfied or regretful about the treatment outcomes[54].

Further analysis revealed that the utilization of social support was most strongly correlated with decision self-efficacy, highlighting the critical role of patients' proactive engagement with available support resources in enhancing self-efficacy. In the context of CRC and chemotherapy, this is especially important, as the complex and prolonged nature of treatment decisions—such as managing fatigue, gastrointestinal distress, and immune suppression—adds an additional layer of uncertainty. These factors make patients more vulnerable to decision regret4]. These findings underscore the need for healthcare providers to not only focus on strengthening patients' external support networks but also to foster their internal psychological resources, particularly self-efficacy. This is essential in helping patients navigate complex, high-stakes treatment decisions, ultimately reducing decision regret.

**Changed:*7 Discussion***

***7.1 Summary of Main Results***

*In this study, the mean decision regret score among CRC patients was 52.52 (SD = 13.40), indicating a moderate level of regret. Decision regret levels varied by across education level, occupation, and monthly personal income. Additionally, higher levels of social support were associated with lower decision regret, and this relationship was partially mediated by decision self-efficacy, which confirmed our hypothesis.*

***7.2 Sociodemographic variables associated with decision regret***

*This study found a significant association between decision regret and income. Consistent with previous research[26,35], patients with poorer economic conditions were more likely to experience regret , primarily due to limited treatment options and restricted access to information[36].Moreover, compared to patients with other types of cancer, those with CRC cancer tend to be more passive in treatment decision-making[37]. Given that chemotherapy often involves prolonged treatment cycles, high costs, and significant side effects, low-income patients are more likely to experience hesitation and post-decision reflection under the dual burden of financial and life stress, which further exacerbates decision regret[38].*

*In addition, decision regret was also significantly associated with occupation. Our findings indicated that unemployed patients reported significantly higher levels of regret compared to employees in public institutions and retirees. This may be related to the reduced financial resources and diminished social support often associated with unemployment. Unemployed patients frequently worry about the economic burden of treatment on their families, the potential decline in quality of life, and even the possibility of incurring debt, making them more vulnerable to self-doubt and regret after treatment[39]. Therefore, clinical practice should address the psychological burden of decision-making among low-income and unemployed patients. Providing financial support, cost counseling, and psychological interventions, in combination with strengthening shared decision-making, may help reduce decision regret, improve adherence, and enhance quality of life.*

*In this study, we found that decision regret among CRC chemotherapy patients significantly decreased with increasing educational level. Specifically, patients with higher education levels reported lower levels of decision regret during the treatment decision-making process. This finding may be attributed to higher health literacy, which enables individuals to better understand complex chemotherapy regimens and more effectively express their personal values, thereby reducing uncertainty and regret[40,41]. In contrast, previous studies have reported differing results. Step et al. [42] have suggested that both patients with lower education levels (associate degree, high school or less) and those with higher education levels (master’s degree or above) are more likely to experience decision regret, whereas those with intermediate education (college) report lower levels of regret, indicating a curvilinear relationship. This phenomenon may be explained by the tendency of highly educated patients to engage in excessive reflection when treatment outcomes are unsatisfactory, thus exacerbating feelings of regret[43].*

***7.3 The Mediating Role of Decision Self-Efficacy***

*Central among theoretical advancements advanced by this investigation lies proof of partial channeling through decision self-efficacy along the pathway joining social support with decision regret encompassing 47% of overall influence. Evidence of this sort lends observational backing to Social Cognitive Theory amid selections for cancer therapies, while broadening earlier inquiries centered largely on prostate and breast malignancy groups [44,45]. Social support appears to exert its protective effect through two distinct pathways. First, consistent with the stress-buffering model, social support provides emotional reassurance and practical assistance, directly alleviating the psychological burden associated with complex CRC treatment decisions. These forms of support may reduce counterfactual thinking and feelings of isolation, thereby decreasing the likelihood of regret[9,46,47]. Second, and more importantly in quantitative terms, social support enhances patients' confidence in their ability to participate meaningfully in treatment decisions. Family members who accompany patients to consultations, help interpret medical information, and offer encouragement directly strengthen patients' perceived control, an essential antecedent of decision self-efficacy[19,48]. In turn, patients with higher decision self-efficacy engage more actively in shared decision-making, ask more questions, and feel a greater sense of ownership over the final choice[23,24]. Even when outcomes are suboptimal, these patients are better equipped to reconcile their decisions with reality, resulting in significantly less regret.*

*The mediating role of decision self-efficacy has important clinical implications. Interventions that focus solely on enhancing external social support are likely to lead to only limited and potentially transient reductions in decision regret unless patients’ internal decision-making competence is also strengthened. This is particularly relevant in colorectal cancer, where treatment decisions typically require patients to weigh survival benefits against substantial, often permanent, quality-of-life consequences[49]. For example, chemotherapy and stoma formation frequently result in changes to physical appearance and bowel function, directly impairing quality of life and heightening patients’ sensitivity to social interactions[50]. Patients with high decision self-efficacy tend to engage more actively and confidently with the healthcare team. They are better able to articulate their values, concerns, and preferences, fostering mutual trust, promoting transparent and open shared decision-making, and reducing uncertainty during the decision process[51,52]. This proactive engagement not only mitigates decisional conflict at the time of choice but also significantly lowers the risk of subsequent regret arising from perceived information deficits or lack of personal involvement[52,53].*

*The study found that decision self-efficacy exhibited different correlation patterns with various types of decision regret. It was more strongly correlated with process regret and option regret, but relatively weakly correlated with outcome regret. This may be due to the fact that treatment outcomes, such as the adverse effects of chemotherapy, are often beyond the patient’s control, which is consistent with previous studies[54]. Even if patients possess strong decision self-efficacy, insufficient health literacy can make it difficult for them to fully understand CRC chemotherapy regimens and subsequent treatments, leading to dissatisfaction or regret regarding treatment outcomes[55].*

*Further analysis revealed that the utilization of social support was most strongly correlated with decision self-efficacy, emphasizing the critical role of patients' proactive engagement with available support resources in enhancing self-efficacy. In the context of CRC and chemotherapy, this is particularly important, as the complex and prolonged nature of treatment decisions—such as managing fatigue, gastrointestinal distress, and immune suppression, adds an additional layer of uncertainty. These factors make patients more vulnerable to decision regret[4]. These findings highlight the need for healthcare providers to not only focus on strengthening patients' external support networks but also on fostering their internal psychological resources, particularly self-efficacy. This is crucial in helping patients navigate complex, high-stakes treatment decisions, ultimately reducing decision regret.*

- Section 3. Response to Reviewer #2:

I would like to express my heartfelt gratitude to you for your careful review and thoughtful feedback on our manuscript. Your professional insights and balanced evaluation—acknowledging the strengths while clearly identifying areas needing refinement—have been immensely valuable in improving the overall quality and clarity of our work. I truly appreciate the time and effort you devoted to assessing our study in such a constructive and encouraging manner.

Your recognition of the study’s theoretical foundation, methodological rigor, and clinical relevance is deeply encouraging to us. At the same time, your critical observations regarding the limitations of the cross-sectional design, the wording of the hypotheses, minor typographical issues, reference inconsistencies, and the need to clarify the timing of variable measurement have provided us with important guidance for strengthening the manuscript. We have carefully addressed each of these issues in the revision.

In particular, your comments regarding the need to exercise caution in interpreting causal relationships, the potential selection bias associated with convenience sampling, and the importance of recommending multi-center longitudinal research in future studies have significantly improved the scientific rigor and interpretative accuracy of the paper. We have revised the Limitations and Discussion sections accordingly to ensure transparency and academic integrity. Additionally, all language errors, incomplete sentences, and reference inconsistencies you highlighted have been systematically corrected to enhance readability and precision.

Below, we provide detailed point-by-point responses to each of your comments. **All modifications have been incorporated into the “Revised Manuscript with Track Changes,” with corresponding revisions clearly indicated for your review.** Your expertise and constructive guidance have greatly enhanced the quality of our work, and we sincerely appreciate your contribution.

★ **1. Cross-sectional design limits causal inference and generalizability**

**Comment:**

**Cross-sectional design is the most significant limitation. Conclusions must be displayed with caution, calling it a preliminary study which cannot be generalized.**

**➦ Response:**

We sincerely thank the reviewer for highlighting this important methodological issue. We fully agree that the cross-sectional design represents the most substantial limitation of our study and that any interpretation involving temporal ordering or causality must be made with great caution.

In response to this valuable comment, we have substantially revised both the Limitations and Conclusion sections to clearly acknowledge that:

1 The cross-sectional nature of the study precludes causal inference, as social support, decision self-efficacy, and decision regret were measured at a single time point.

2 The observed associations should be interpreted as correlational rather than directional, and the mediation pathway represents only a statistical rather than causal mediation model.

3 Our findings should be considered preliminary, serving primarily as hypothesis-generating evidence rather than definitive conclusions.

4 Generalizability is limited, given the single-center setting, convenience sampling, and the demographic profile of our participants.

We also added explicit statements that confirm the need for longitudinal or multi-wave designs in future research. These revisions are now reflected in the manuscript as follows (with modifications highlighted in blue)

➦**Comparison before and after modification**

**Before modification：Limitations**

This study has several limitations. First, the cross-sectional design prevents causal inferences and may introduce recall bias. The sample was recruited from a single tertiary hospital in Inner Mongolia, limiting the generalizability of the findings to broader CRC populations. This was due to time and budget constraints, which restricted the study to a single site. Additionally, the use of convenience sampling, while efficient, may have reduced the representativeness of the sample. Despite these limitations, the study provides valuable insights, and future research with larger, more diverse samples, and longitudinal or interventional designs, is needed to validate and extend these findings.

**Conclusion**

The level of decision regret among CRC patients was significantly associated with occupation, monthly income, and educational attainment. In addition, both social support and decision self-efficacy were identified as negative predictors of decision regret, with decision self-efficacy mediating the impact of social support on decision regret. These findings suggest that, in the context of CRC care, greater emphasis should be placed on developing targeted interventions and strengthening social support systems to effectively alleviate patients’ decision regret.

**Changed:*8 Limitations***

*Several constraints characterize this inquiry. Primarily, adoption of a cross-sectional framework obstructs derivation of causality and curtails capacity to delineate chronological linkages connecting decision regret, decision self-efficacy, and social support, all the while fostering susceptibility to retrospective distortion. Next, recruitment occurred solely from one advanced-care facility located in Inner Mongolia, a circumstance compounded by reliance on opportunistic enrollment that could curtail applicability of outcomes beyond this locale and engender preferential enrollment distortions, given that individuals under tertiary oversight typically encounter amplified availability of aids and backing relative to counterparts in alternative therapeutic contexts. Even so, substantive initial insights emerge from this work into mental processes shaping choices for chemotherapy among those afflicted with CRC. Future research should employ multi-center, longitudinal designs to strengthen external validity and clarify the temporal pathways among the study variables.*

***10 Conclusion***

*The level of decision regret among CRC patients was significantly associated with occupation, monthly income, and educational attainment. Additionally, both social support and decision self-efficacy were negatively associated with decision regret, with decision self-efficacy mediating the effect of social support on decision regret. These findings suggest that, in the context of CRC care, greater emphasis should be placed on developing targeted interventions and strengthening social support systems to effectively alleviate patients' decision regret.*

We deeply appreciate the reviewer’s insightful recommendation, which has significantly enhanced the clarity, rigor, and transparency of our manuscript. Should further specification be required, we are fully willing to revise accordingly.

★ **2. Minor typos / incomplete sentences**

**Comment:**

**There are minor typos and incomplete sentences (e.g., “accompanied by (3)…”).**

**➦ Response:**

We sincerely thank the reviewer for pointing out the minor typographical errors and incomplete sentences in the manuscript. We appreciate this careful attention to detail.

In response to this comment, we conducted a thorough, line-by-line proofreading of the entire manuscript. All typographical issues, incomplete sentences, punctuation inconsistencies, and formatting problems (including the example noted by the reviewer, “accompanied by (3)…”) have now been corrected. Revised expressions have been clearly marked in the version with track changes.

➦**Comparison before and after modification**

**Before modification：**CRC has thus become a major public health concern and a serious threat to human health. Currently, chemotherapy remains a cornerstone in the treatment of CRC , aiming to eradicate residual disease after surgery and improve the cure rate. accompanied by[3].

**Changed：***Figures presented in the Global Cancer Statistics from the International Agency for Research on Cancer (IARC)[2], reveal about 1.926 million instances of initial CRC detection in 2022, which form 9.6% of overall cancer detections while fatalities from this disease make up 9.3% of cancer- induced mortalities. These figures underscore CRC as a major global public health burden. Currently, chemotherapy remains a key component of CRC treatment, aiming to eliminate residual disease after surgery and improve overall survival[3].*

We are grateful for this helpful observation, which has improved the accuracy and readability of the manuscript. If the reviewer identifies any additional areas that would benefit from further refinement, we would be more than willing to revise them.

★ **3. Citation mismatch (“Lee & Bryant-Lukosius (14)”)**

**Comment:**

**The citation “Lee & Bryant-Lukosius (14)” does not match the reference list.**

**➦ Response:**

We sincerely thank the reviewer for drawing our attention to this citation inconsistency. We fully agree that accuracy and consistency between in-text citations and the reference list are essential for clarity and scholarly rigor.

In response to your comment, we carefully re-checked all citations throughout the manuscript, with particular attention to the reference noted (“Lee & Bryant-Lukosius (14)”). We identified the mismatch and have now corrected the in-text citation to ensure it accurately corresponds to the correct entry in the reference list. Additionally, we performed a comprehensive audit of all other citations to verify consistency in numbering, author names, and publication details. All corrections have been clearly highlighted in the revised manuscript with track changes.

➦**Comparison before and after modification**

**Before modification：**However, research by Lee & Bryant-Lukosius[14]. found that approximately 64% of CRC patients exhibit significant deficiencies in decision self-efficacy, an issue that urgently needs to be addressed. Notably, social support plays a crucial role in enhancing decision self-efficacy. Research shows that social support can enhance an individual's cognitive evaluation and sense of control, thereby promoting the improvement of their self-efficacy[14].

**Changed：***However, Lee and Bryant-Lukosius reported that approximately 64% of patients with CRC have marked deficiencies in decision self-efficacy [15]*

We are grateful for this valuable observation, which has improved the precision and reliability of our referencing. Should the reviewer identify any further discrepancies, we would be happy to revise them promptly.

★ **4. Missing full reference for “Temitope G”**

**Comment:**

**The cited study by “Temitope G” lacks complete bibliographic information.**

**➦ Response:**

We sincerely appreciate the reviewer for pointing out the incomplete bibliographic information for the citation attributed to “Temitope G.” Accurate and complete referencing is essential to ensure traceability and scholarly integrity, and we are grateful for the opportunity to correct this oversight.

In response to your comment,we have now retrieved the complete bibliographic details of the cited work and updated both the in-text citation and the reference list accordingly. All corrections are clearly marked in the revised manuscript using track changes.

Furthermore, to avoid similar issues, we rechecked the entire reference list to ensure that all citations are complete, consistent, and aligned with journal formatting requirements.

➦**Comparison before and after modification**

**Before modification：**In a prospective cohort study conducted by Temitope G, approximately 31% of rectal cancer patients were classified as having high decision regret at one year of follow-up after surgery.

**Changed：***In a prospective cohort study conducted by Temitope G, approximately 31% of rectal cancer patients were classified as having high decision regret at 1-year of follow-up after surgery[11].*

[11]Joshua TG, Robitaille S, Paradis T, et al. Decision-making preferences and regret in rectal cancer patients undergoing restorative proctectomy: A prospective cohort study. Surgery. 2024;176(4):1065-1071. doi:10.1016/j.surg.2024.05.019

We thank the reviewer again for this helpful observation, which has strengthened the accuracy and professionalism of our manuscript. Should additional adjustments be needed, we will be happy to revise further.

**★ 5. Hypotheses require rephrasing to improve clarity**

**Comment:**

**Hypotheses are hard to comprehend and weakly worded (e.g., “Patients’ social support and self-efficacy may account for …”).**

**➦ Response:**

We sincerely thank the reviewer for this valuable comment regarding the clarity and wording of the study hypotheses. We fully agree that clearly articulated and precisely stated hypotheses are essential for enhancing the conceptual rigor and readability of the manuscript. Your observation has been extremely helpful in prompting us to refine and strengthen this section.

In response to your suggestion, we have thoroughly revised the Aims and Hypotheses subsection to ensure that the hypotheses are concise, direct, and aligned with both the theoretical framework and the analyses conducted. Specifically, we removed tentative or ambiguous phrasing such as “may account for,” and reformulated the hypotheses to express clear directional relationships grounded in the Stress-Buffering Model and Social Cognitive Theory.

The revised hypotheses now read as follows:

*(H1) There is a negative association between social support and decision regret; (H2) There is a negative association between decision self-efficacy and decision regret; and (H3) Decision self-efficacy mediates the association between social support and decision regret.*

These revisions improve clarity, ensure conceptual alignment with the mediation model, and present the hypotheses in a more assertive and scientifically appropriate manner. The updated hypothesis section is now clearly marked in the revised manuscript with track changes.

We are grateful for this insightful recommendation, which has substantially strengthened the coherence and communicative precision of the manuscript. Should further clarification be helpful, we would be pleased to make additional refinements.

**★ 6. No description of measurement timing during chemotherapy cycles**

**Comment:**

**There is no mention of measurement timing of different variables across cycles.**

**➦ Response:**

We sincerely thank the reviewer for highlighting the important issue regarding the timing of measurements across chemotherapy cycles. This is an excellent point, and we agree that specifying when each variable was assessed is essential for ensuring transparency and for accurately interpreting the findings.

In response to your comment, we have added a clear and detailed description of the measurement timing to the Methods section. Specifically, this information has been incorporated into Section **“3.2 Setting, Participants, and Procedure.”** In the revised manuscript, we now clarify that all study variables were assessed at a single time point during each participant’s scheduled chemotherapy visit. Participants completed the questionnaires before receiving their chemotherapy infusion on the day of data collection. As patients were at different stages of their chemotherapy courses, the measures were collected cross-sectionally and were not repeated across cycles. Additionally, the number of completed chemotherapy cycles was documented as a clinical variable to account for treatment-stage differences.The revised text added to the manuscript is as follows (now marked in the revised version):

***“****All study variables were measured at a single time point during the patient’s current chemotherapy visit. Participants completed the questionnaires before receiving their scheduled chemotherapy cycle on that day. Although patients were at different stages of chemotherapy , all measures were collected cross-sectionally and were not repeated across cycles. The number of completed chemotherapy cycles was recorded as a clinical variable.****”***

This clarification ensures that readers can better understand the context of data collection and strengthens the methodological transparency of the study.

We sincerely appreciate the reviewer’s thoughtful observation and guidance. Your comment has helped us improve the completeness and clarity of the Methods section, and we remain grateful for your constructive feedback.

**★ 7. Convenience sampling increases risk of selection bias**

**Comment:**

**Convenience sampling may introduce selection bias (e.g., tertiary hospital patients may have better support).**

**➦ Response:**

We sincerely appreciate the reviewer’s thoughtful comment regarding the potential selection bias introduced by the use of convenience sampling. Your observation is highly valid, particularly given that the study was conducted in a tertiary hospital setting, where patients may have better access to medical resources and stronger family or institutional support compared with those in primary or rural settings.

In response to your comment, we have expanded the Limitations section to explicitly address this issue. The revised manuscript now acknowledges that convenience sampling may limit the representativeness of the study sample and may introduce systematic bias. Specifically, patients treated in tertiary hospitals often benefit from more comprehensive care teams, higher health literacy, and stronger social support networks, which may influence both their decision self-efficacy and decision regret. This means that the findings may not fully generalize to patients in lower-resourced settings or different healthcare environments.

To address this concern transparently, we added the following content to the Limitations section (highlighted in blue in the revised manuscript):

“*Several constraints characterize this inquiry. Primarily, adoption of a cross-sectional framework obstructs derivation of causality and curtails capacity to delineate chronological linkages connecting decision regret, decision self-efficacy, and social support, all the while fostering susceptibility to retrospective distortion. Next, recruitment occurred solely from one advanced-care facility located in Inner Mongolia, a circumstance compounded by reliance on opportunistic enrollment that could curtail applicability of outcomes beyond this locale and engender preferential enrollment distortions, given that individuals under tertiary oversight typically encounter amplified availability of aids and backing relative to counterparts in alternative therapeutic contexts. Even so, substantive initial insights emerge from this work into mental processes shaping choices for chemotherapy among those afflicted with CRC. Future research should employ multi-center, longitudinal designs to strengthen external validity and clarify the temporal pathways among the study variables.*”

By incorporating this revision, we aim to provide a more accurate and balanced interpretation of our findings and clearly communicate the boundaries of their generalizability. We sincerely appreciate the reviewer’s insightful suggestion, which has strengthened the methodological transparency and scientific rigor of our manuscript.

**★ 8. Future directions should emphasize multi-center, longitudinal study**

**Comment:**

**Future directions need multi-center longitudinal design.**

**➦ Response:**

We sincerely thank the reviewer for this valuable suggestion regarding the need for multi-center longitudinal research in future studies. We fully agree that extending the current research design will substantially strengthen the evidence base and improve the generalizability and causal interpretability of the findings.

In response to your comment, we have revised the Limitations section to explicitly emphasize the importance of conducting multi-center longitudinal studies. Specifically, we now acknowledge that the current single-center, cross-sectional design limits both causal inference and the representativeness of the sample, and that future research should incorporate a broader and more diverse patient population across multiple clinical settings.

The following sentences have been added to the Future Directions subsection (highlighted in blue in the revised manuscript):

“*Several constraints characterize this inquiry. Primarily, adoption of a cross-sectional framework obstructs derivation of causality and curtails capacity to delineate chronological linkages connecting decision regret, decision self-efficacy, and social support, all the while fostering susceptibility to retrospective distortion. Next, recruitment occurred solely from one advanced-care facility located in Inner Mongolia, a circumstance compounded by reliance on opportunistic enrollment that could curtail applicability of outcomes beyond this locale and engender preferential enrollment distortions, given that individuals under tertiary oversight typically encounter amplified availability of aids and backing relative to counterparts in alternative therapeutic contexts. Even so, substantive initial insights emerge from this work into mental processes shaping choices for chemotherapy among those afflicted with CRC. Future research should employ multi-center, longitudinal designs to strengthen external validity and clarify the temporal pathways among the study variables.*”

These revisions provide a clearer roadmap for advancing the field and reflect the reviewer’s insightful recommendations. We truly appreciate your constructive feedback, which has significantly improved the depth and forward-looking perspective of our manuscript.
